# Supplementary material for: Dynamic Transcriptional Landscape of Grass Carp (Ctenopharyngodon idella) Reveals Key Transcriptional Features Involved in Fish Development
Source: Int J Mol Sci. 2022 Sep 30;23(19):11547. doi: 10.3390/ijms231911547 (PMC9569805; doi:10.3390/ijms231911547)
Supplement: Supplementary file 1 [file ijms-23-11547-s001.zip › supp_figures.pdf]

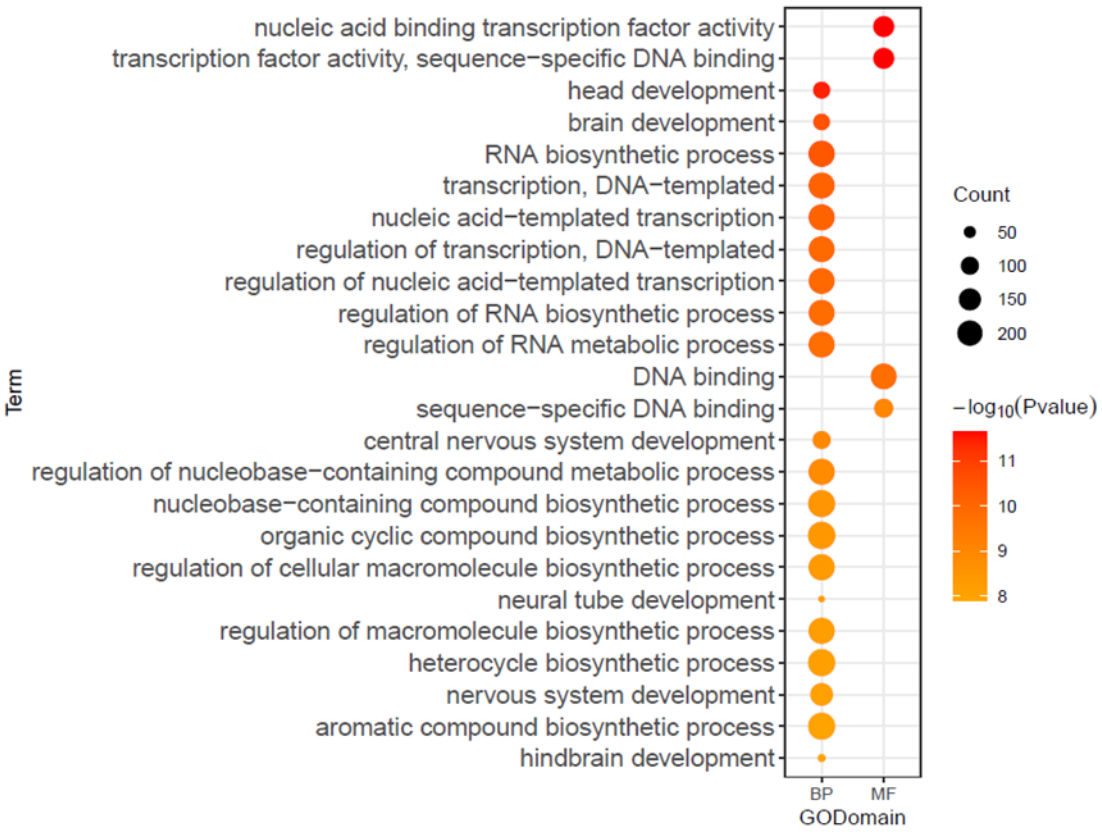

Supplemental Figure S1. GO enrichment results of PC2-related genes in principle components analysis (PCA) on all samples (Top 24).

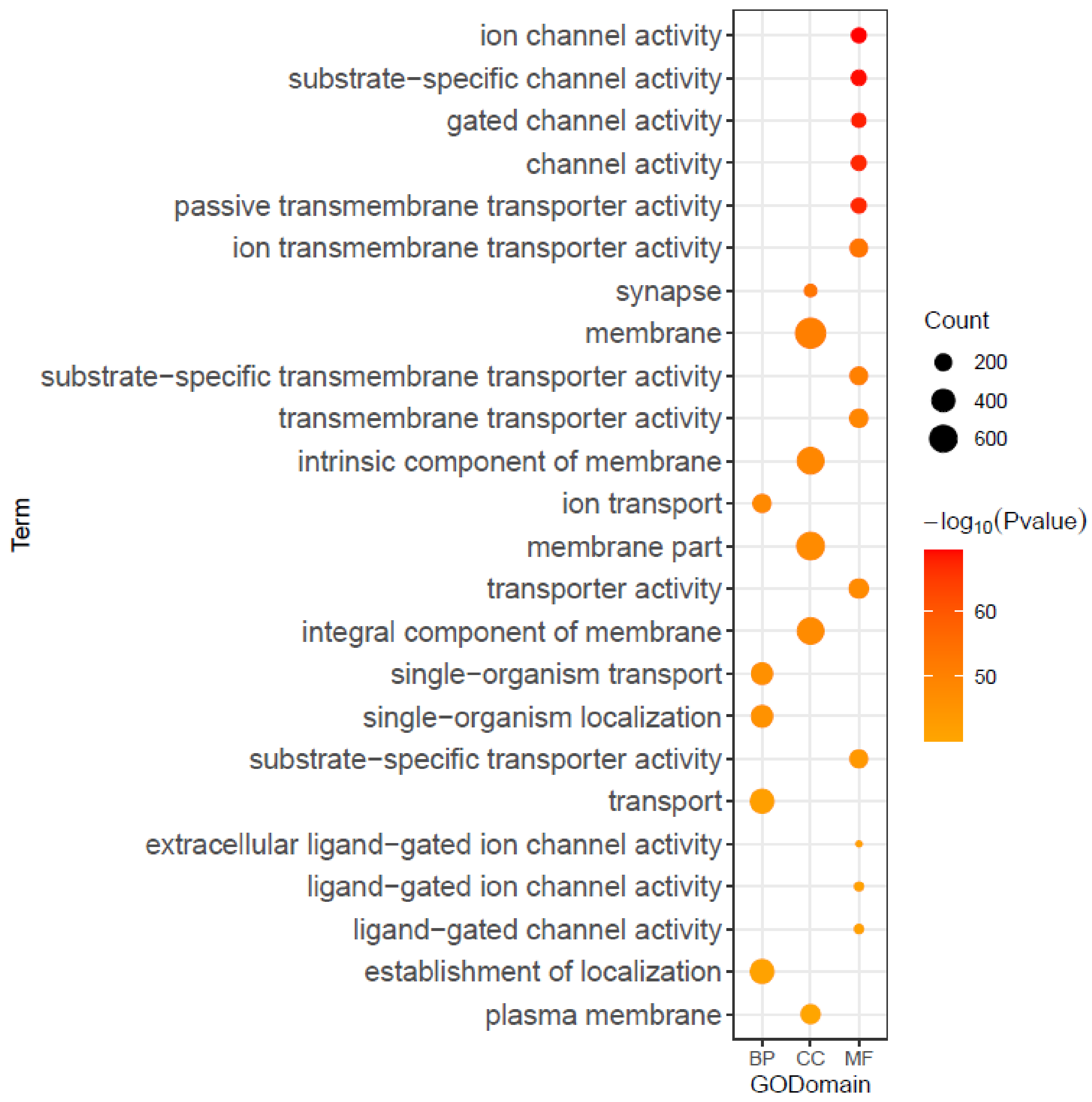

Supplemental Figure S2. GO enrichment results of PC3-related genes in PCA analysis on all samples (Top 24).

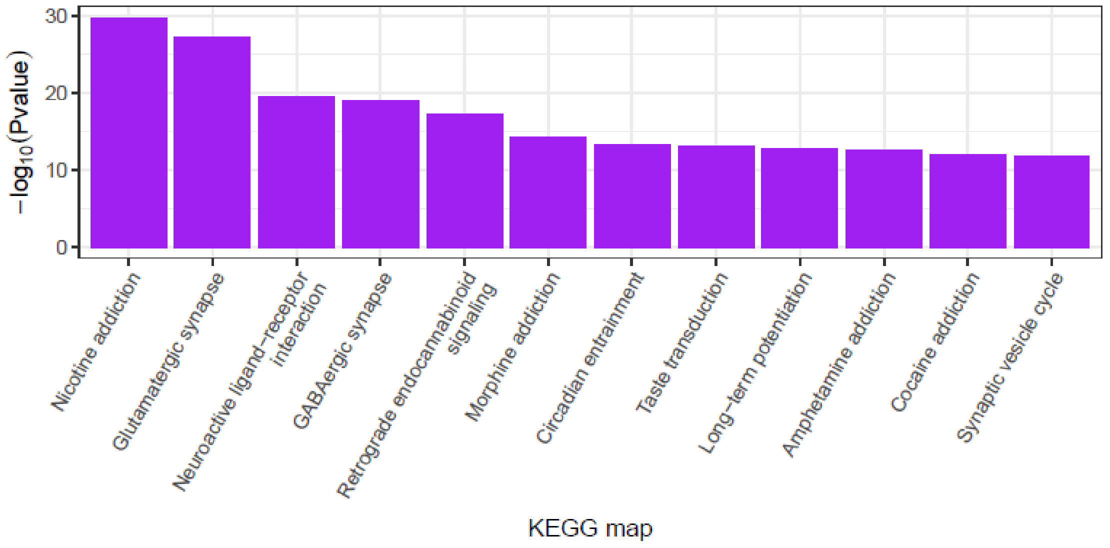

Supplemental Figure S3. KEGG enrichment results of PC3-related genes in PCA analysis on all samples (Top 12).

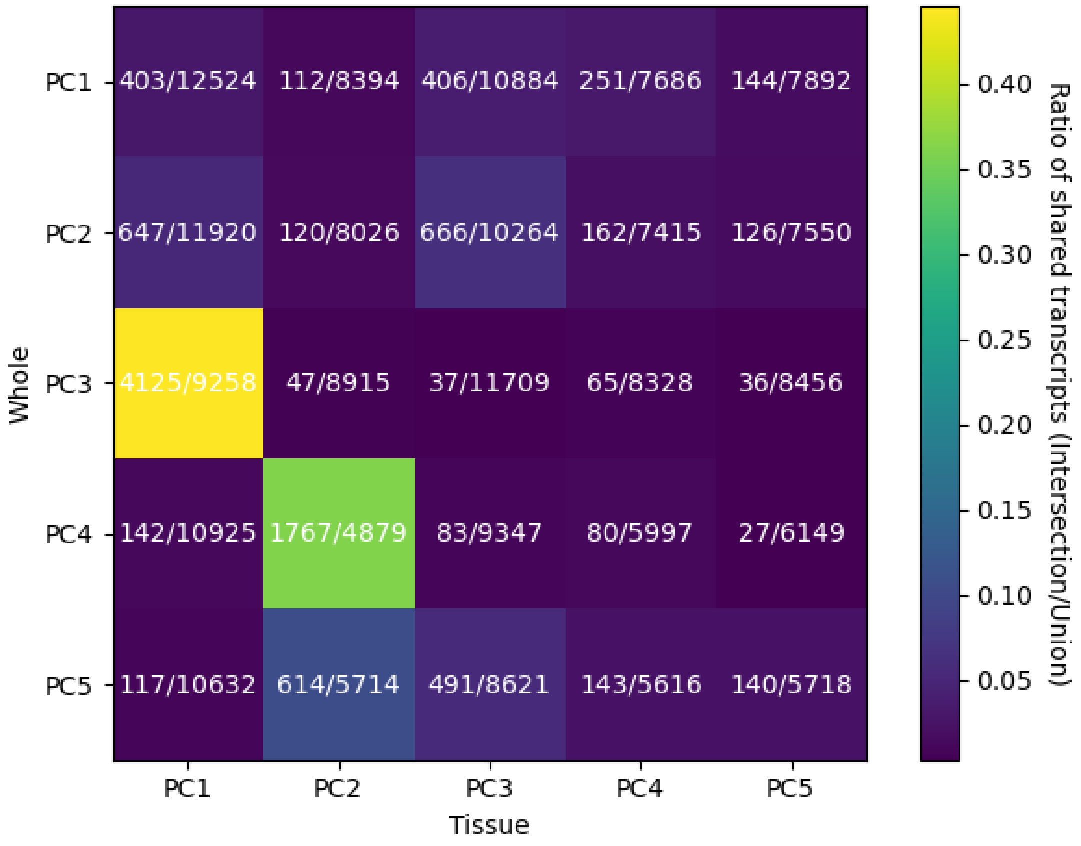

Supplemental Figure S4. Similarity of principle components between in PCA analysis on all samples and in PCA analysis on juvenile samples. The ratio of shared transcripts was calculated by dividing the element number in the intersection of PC-related transcripts by

the element number in the union of PC-related transcripts. Rows are principle components in analysis on all samples, while columns are principle components in analysis on juvenile samples.

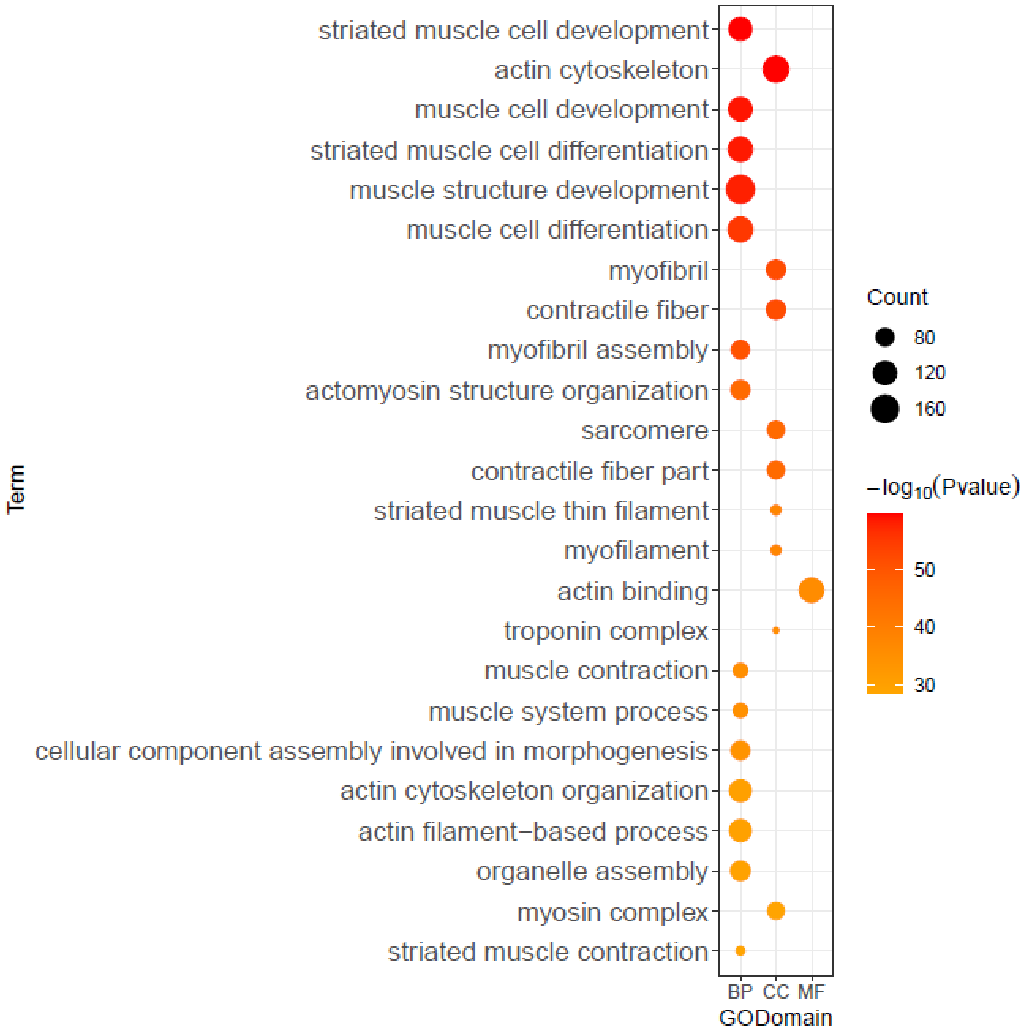

Supplemental Figure S5. GO enrichment results of PC2-related genes in PCA analysis on juvenile samples (Top 24).

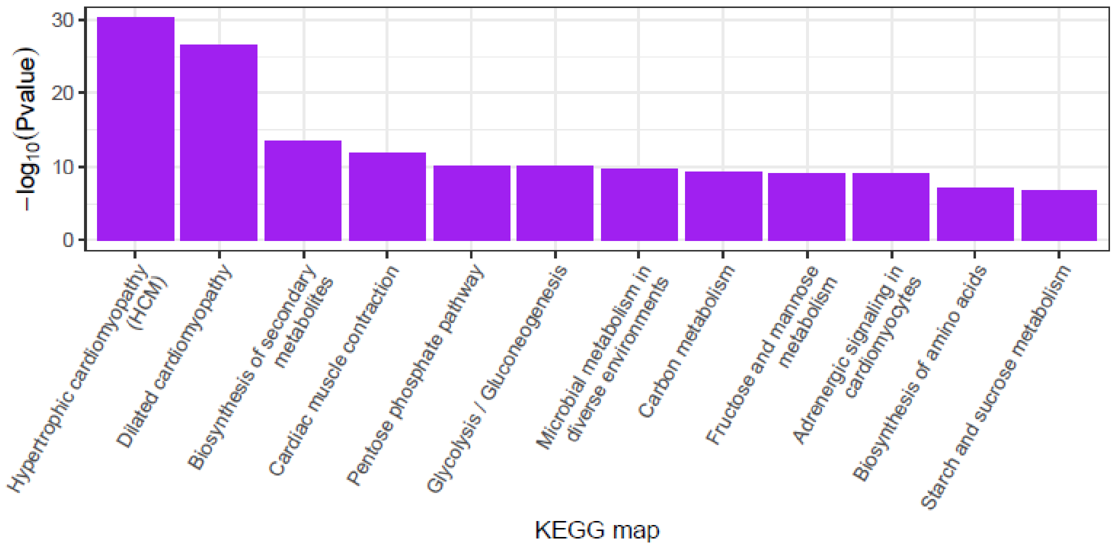

Supplemental Figure S6. KEGG enrichment results of PC2-related genes in PCA analysis on juvenile samples (Top 12).

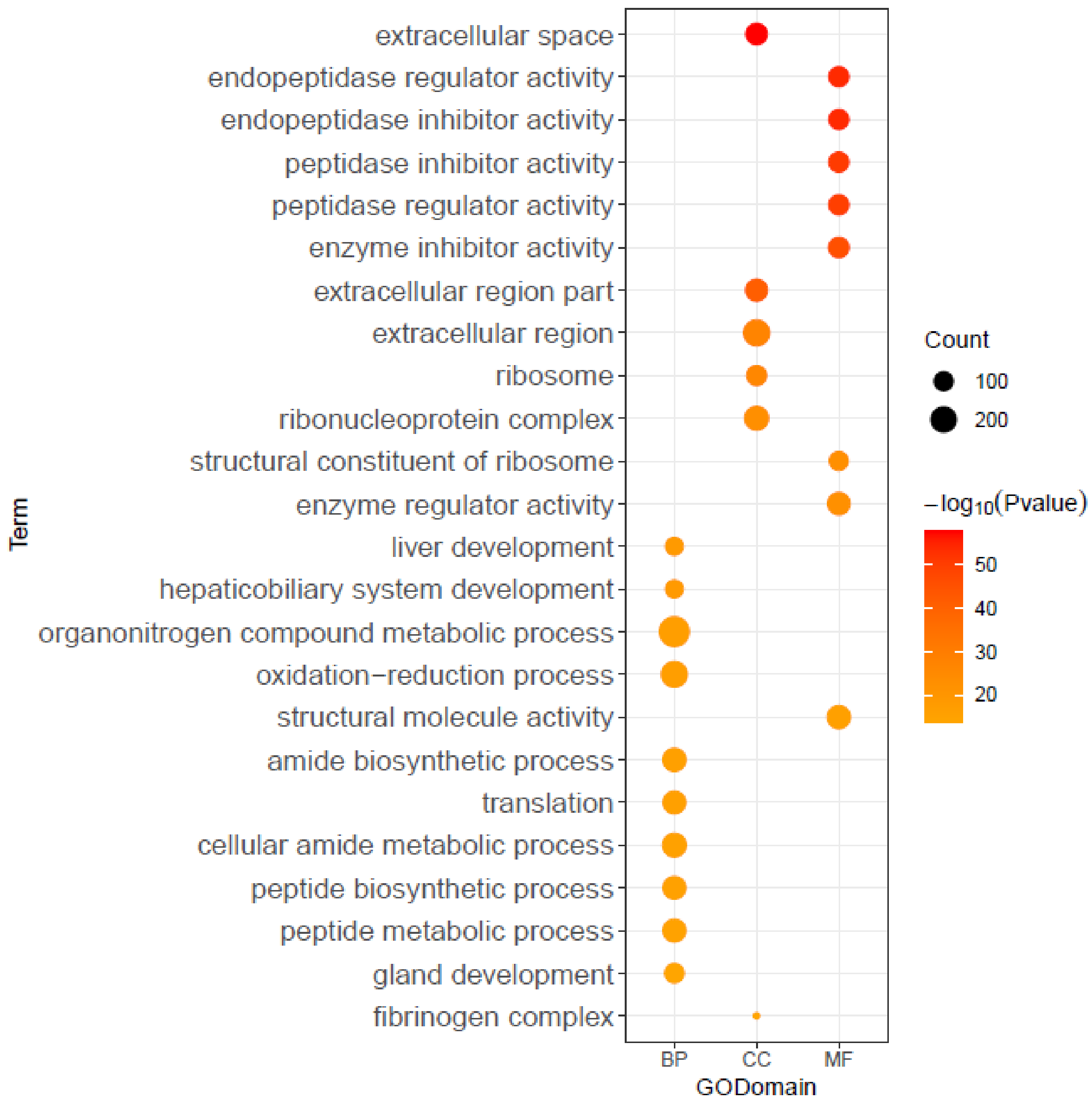

Supplemental Figure S7. GO enrichment results of PC3-related genes in PCA analysis on juvenile samples (Top 24).

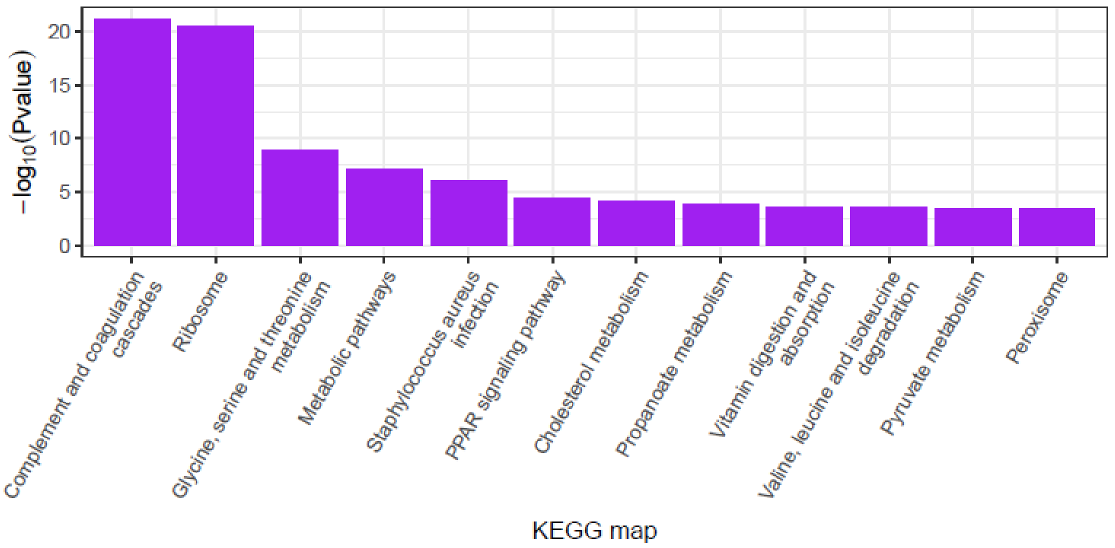

Supplemental Figure S8. KEGG enrichment results of PC3-related genes in PCA analysis on juvenile samples (Top 12).

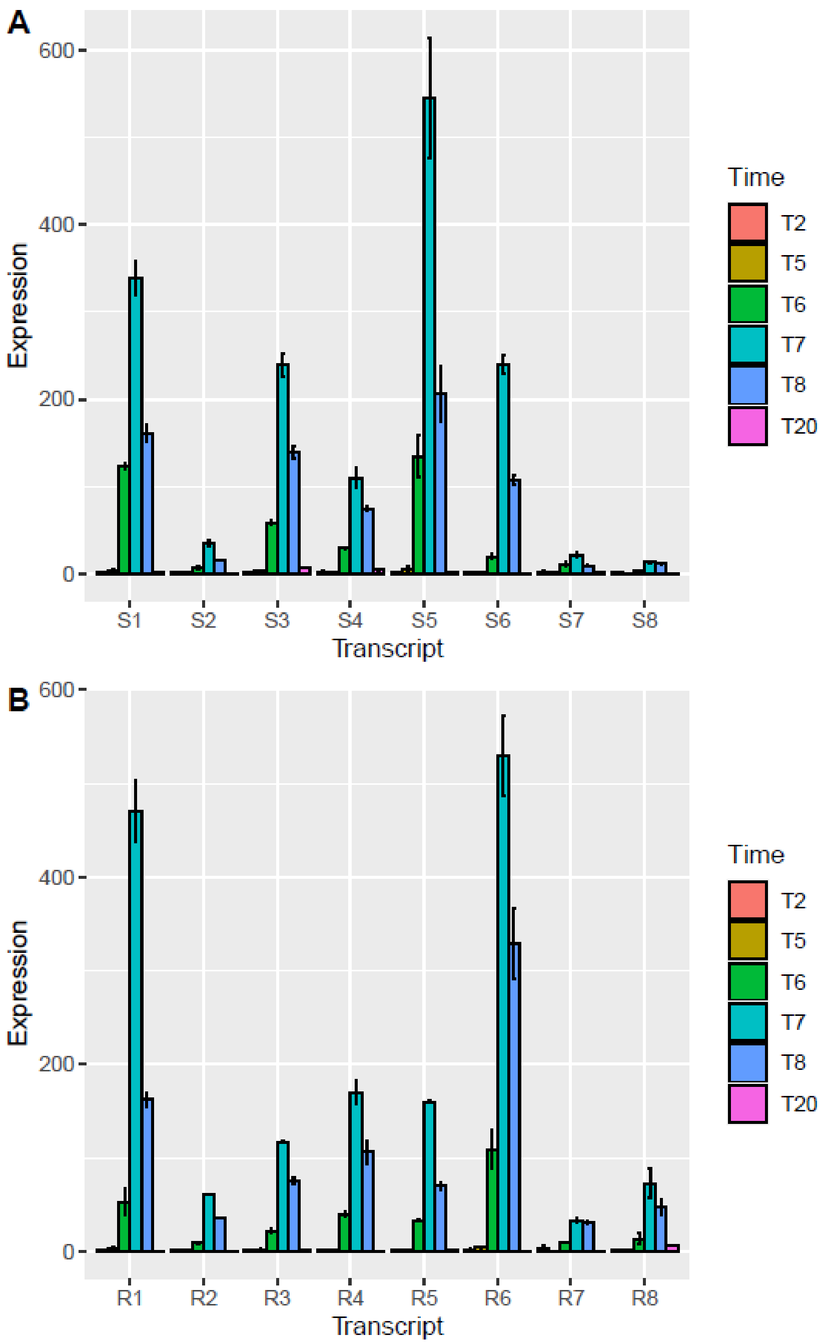

Supplemental Figure S9. The expression level of 16 randomly selected genes measured by using RT-qPCR (ΔΔCt). The RPS27 gene (small subunit ribosomal protein S27e, gene ID is CIWT.8640) was selected as the internal reference. See Methods section for more details. (A) The expression of eight randomly selected short interspersed nuclear element (SINE) genes that belong to the rnd-3_family-293 family. The gene IDs of S1 - S8 are CIWT.14110, CIWT.8438, CIWT.18867, CIWT.14931, CIWT.9147, CIWT.9101, CIWT.7517 and CIWT.31526 respectively; (B) The expression of eight randomly selected

Motif-related genes, which have the same combination of motif 1 and motif 5 as rnd-3_family-293 gene do. The gene IDs of R1 - R8 are CIWT.15163, CIWT.14112, CIWT.17561, CIWT.29641, CIWT.2499, CIWT.30877, CIWT.15487 and CIWT.750 respectively.

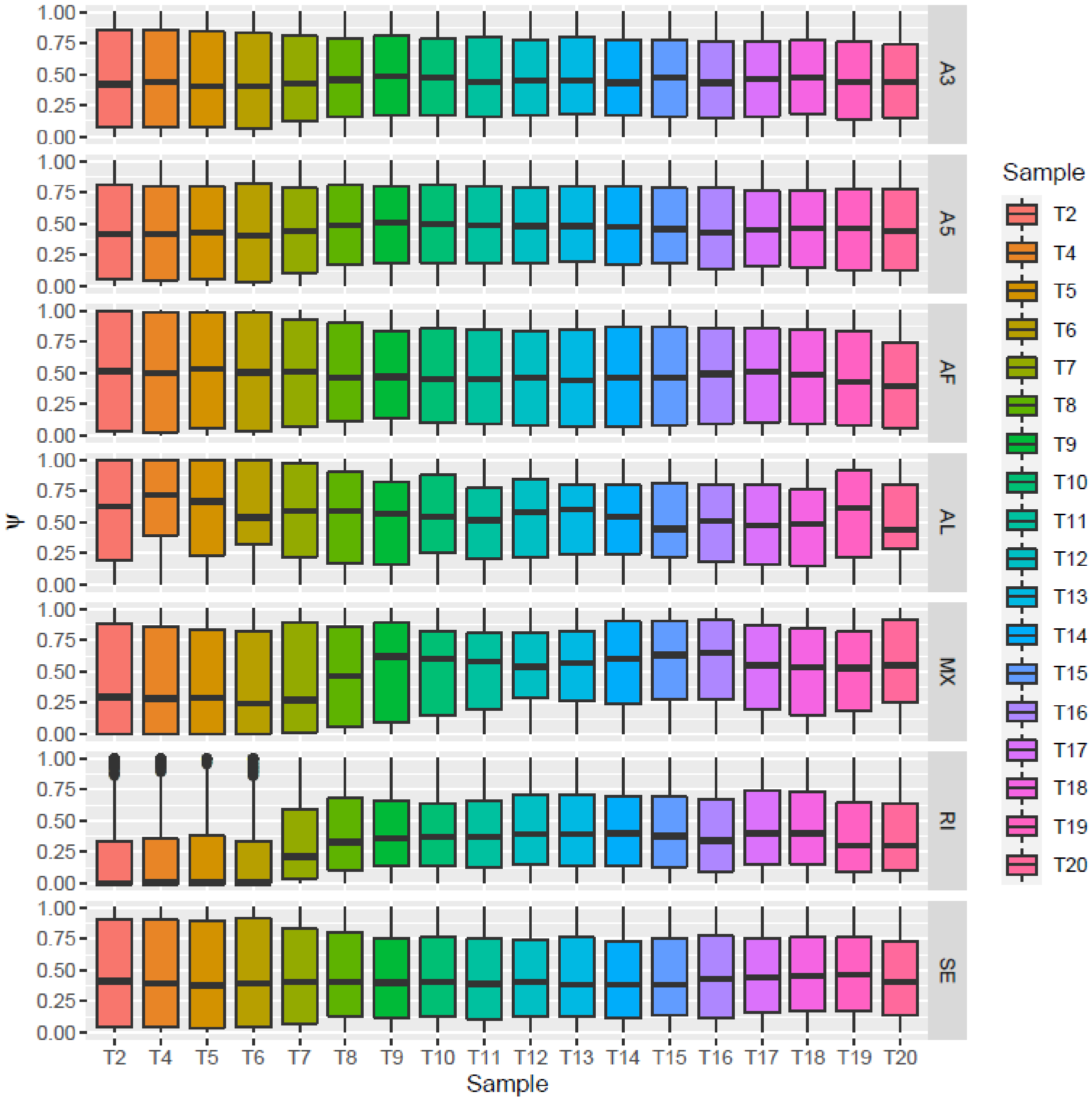

Supplemental Figure S10. Distribution of Ψ value in samples from T2 to T20.

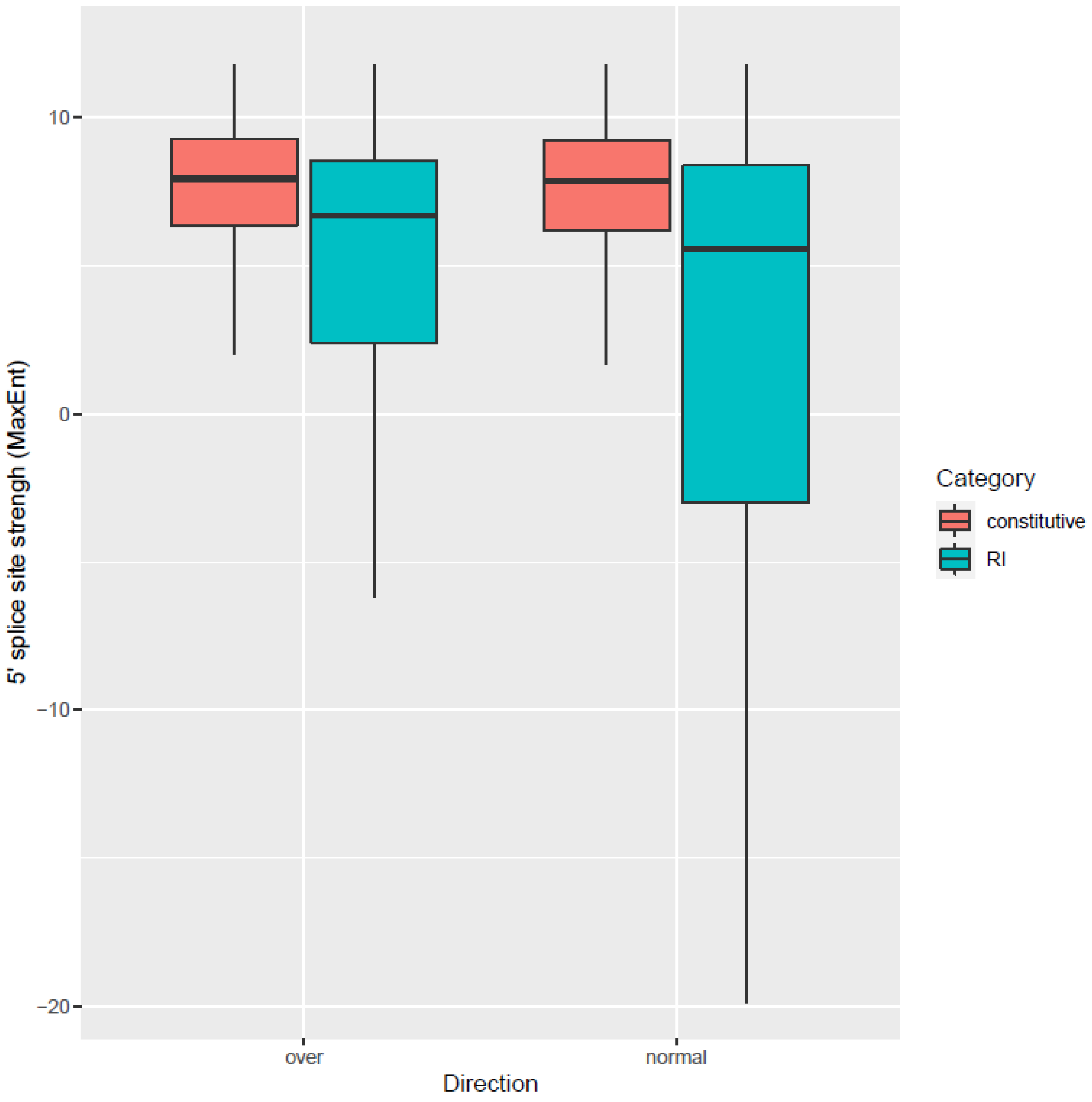

Supplemental Figure S11. Strength of 5' splicing site (MaxEnt) of genes that have retained intron (RI) alternative splicing events. The strength of constitutive introns and retained intron (RI) introns was calculated separately. Based on the difference of the Ψ values of RI gene between time T7 and time T6 (Ψ(T7) − Ψ(T6)), the events with a difference bigger than 0.07 were classified as an "over" event and referred to as "over" later, while the events with a difference smaller than 0.07 were defined as a "normal" event and referred to as "normal" hereafter.

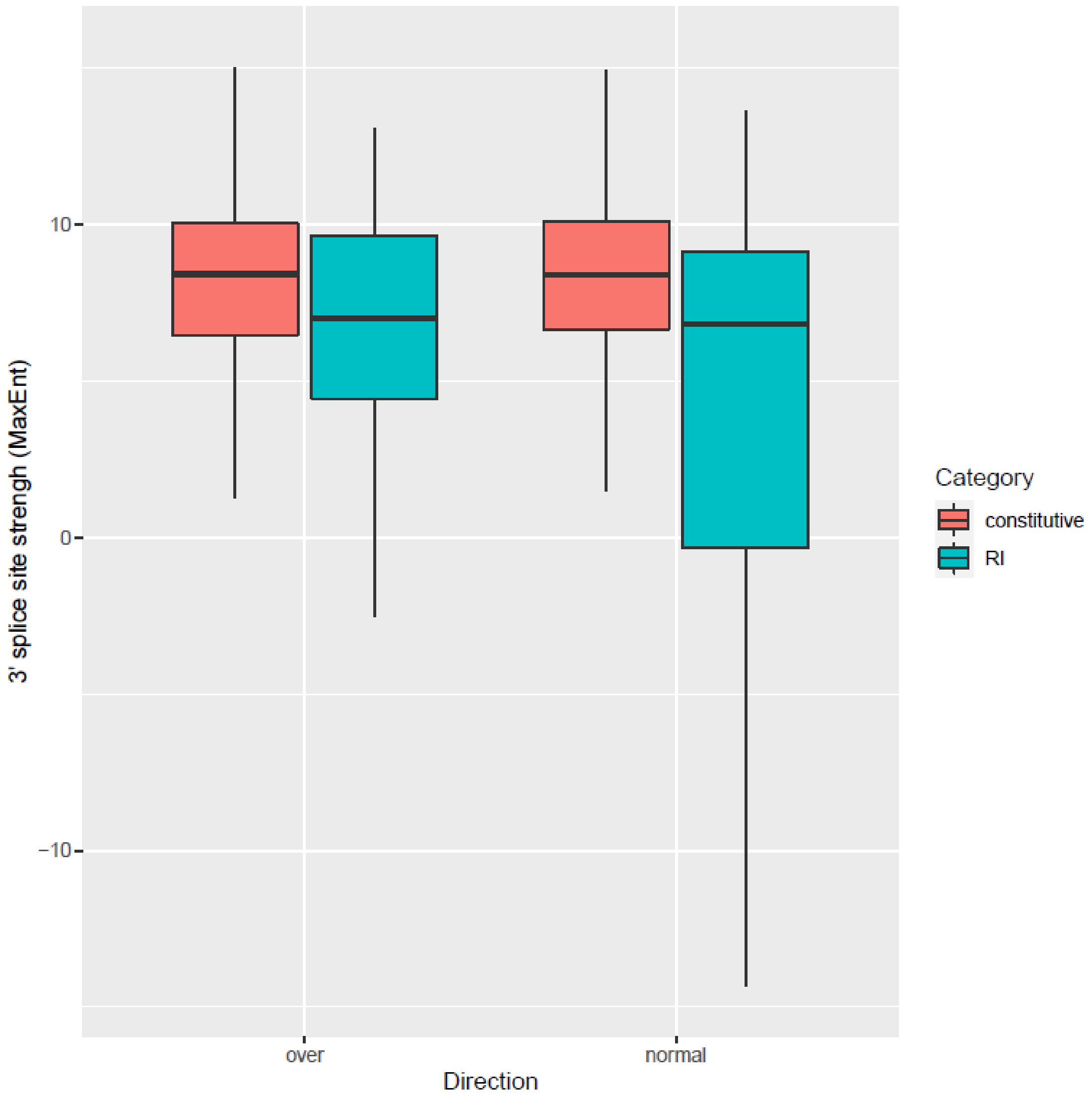

Supplemental Figure S12. Strength of 3' splicing site (MaxEnt) of genes that have RI alternative splicing events. The strength of constitutive introns and retained intron (RI) introns was calculated separately.

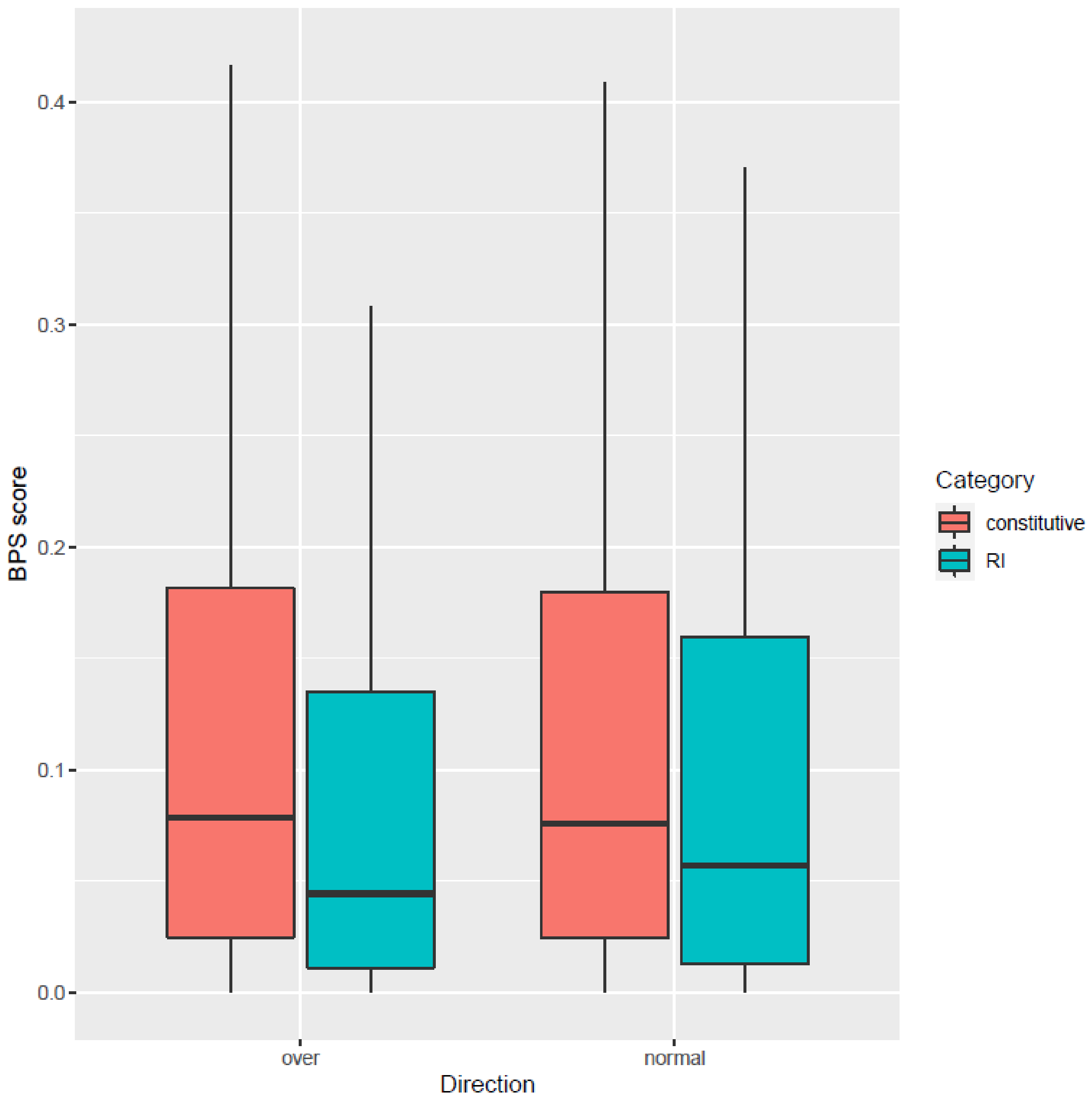

Supplemental Figure S13. Branch points scores (BPS) of genes that have RI alternative splicing events (computed by BPP). The BPS of constitutive introns and retained intron (RI) introns was calculated separately.

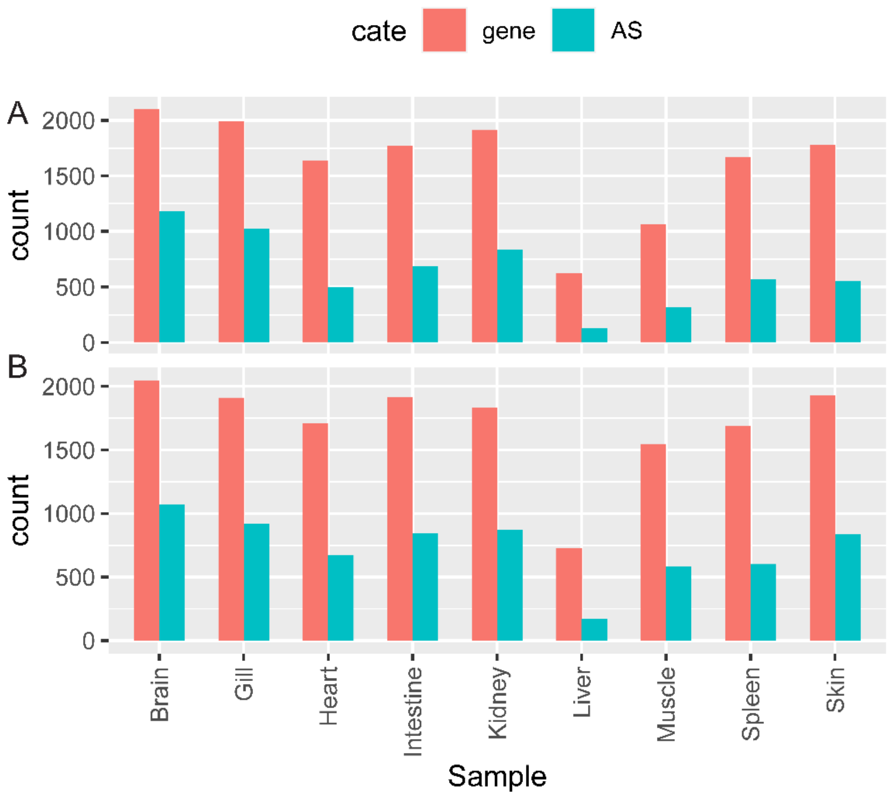

Supplemental Figure S14. The number of alternative splicing events is positively correlated with the number of expressed genes. (A) 134 dpf (R^2=0.91); (B) 197dpf (R^2=0.82).

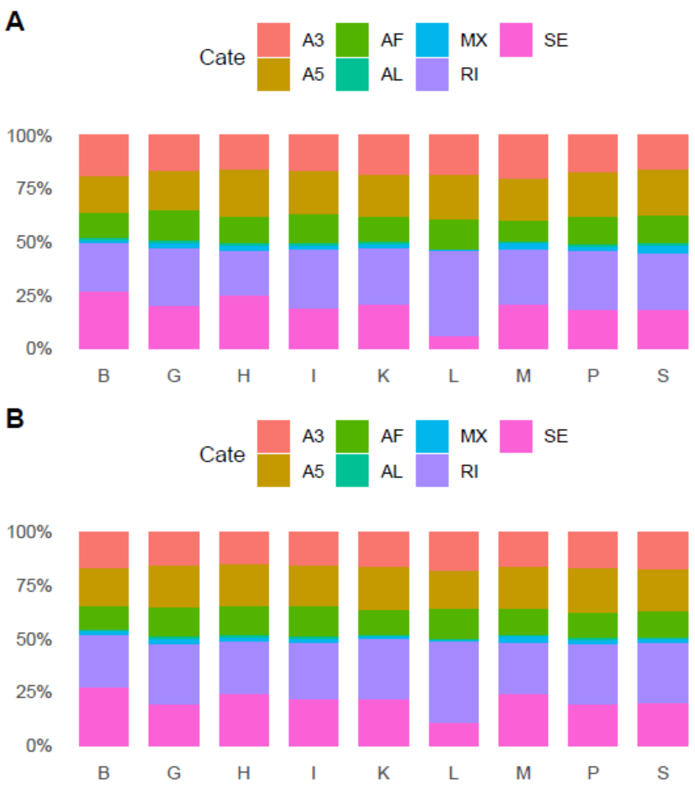

Supplemental Figure S15. Distribution of alternative splicing event types expressed in nine grass carp tissues at (A) 134 days post fertilization (dpf) and (B) 197 dpf.

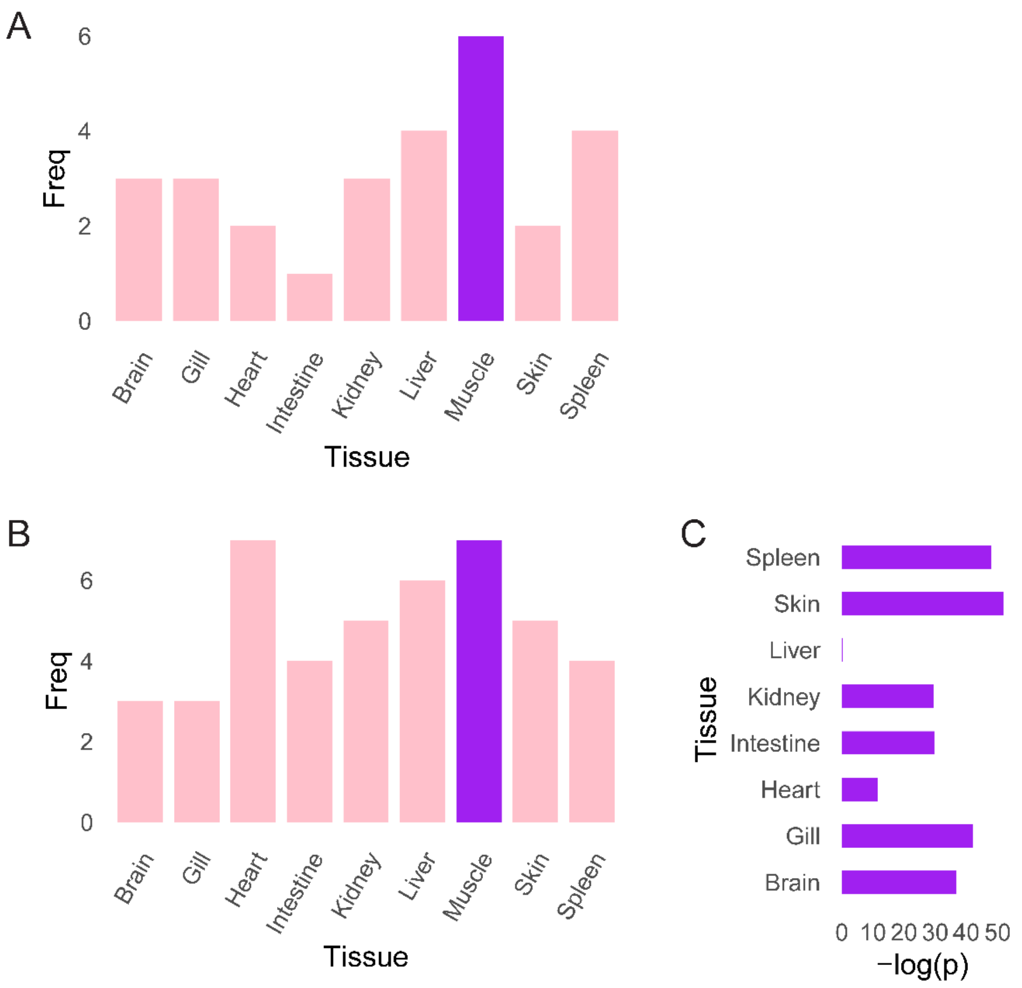

Supplemental Figure S16. An examination of the alternative splicing events among tissues. (A) The number of tissues that are statistically different from one tissue ($P < 0.05$). To undertake pairwise comparisons of the distribution of values Ψ across tissues, a Wilcoxon signed-rank test was utilized; (B) The number of significant differences that arise when one tissue is compared to other tissues according to Ψ values of RI events (Wilcoxon signed-rank test, $P < 0.05$); (C) The significance of the difference in value between muscle tissue and other tissues in RI splicing events was investigated.

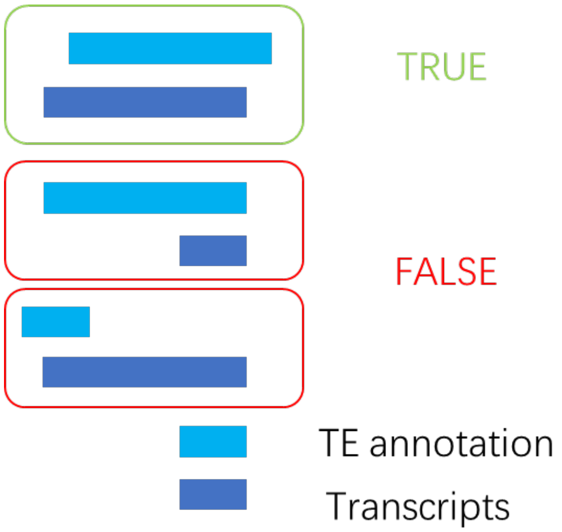

Supplemental Figure S17. Scheme shows the principles of identification of expressed transposable elements.
